# Associations of cannabis, alcohol, and tobacco use combinations with sleep health

**DOI:** 10.1016/j.abrep.2026.100680

**Published:** 2026-02-21

**Authors:** David A. Reichenberger, Joey Hebl, Steven A. Shea, Nicole P. Bowles

**Affiliations:** a Oregon Institute of Occupational Health Sciences, Oregon Health & Science University, Portland, OR, USA; b Knight Cardiovascular Institute, Oregon Health & Science University, Portland, OR, USA

**Keywords:** Multidimensional sleep health, Sleep, Cannabis, Simultaneous alcohol and marijuana, Polysubstance use, Cannabis dependence, Substance use

## Abstract

**Objectives:**

Cannabis is often used alongside other substances, including cigarettes and alcohol. The objective of this study was to identify how the combination of these substances may affect sleep health.

**Methods:**

Data from an online, national survey of 518 adults (35.2 ± 13.4 years old; 65% female) were analyzed. Respondents reported their cannabis, cigarette, and alcohol use. Hazardous cannabis use was assessed using the Cannabis Use Disorder Identification Test – Revised (CUDIT-R). Sleep, insomnia, and daytime sleepiness were assessed using the Pittsburgh Sleep Quality Index (PSQI), Insomnia Severity Index (ISI), and Epworth Sleepiness Scale (ESS), respectively. Sleep health was assessed using PSQI and ISI items about sleep quality, satisfaction, trouble staying awake, bedtime, waketime, sleep efficiency, and duration. Linear regression models examined associations of CUDIT-R and substance use with sleep scales and the individual sleep items, adjusting for sociodemographic factors.

**Results:**

One-quarter used only cannabis, 45% alcohol and cannabis, 12% cigarettes and cannabis, and 19% all three substances (polysubstance use). Average scores were 10.4 ± 5.7 on the CUDIT-R, 8.0 ± 4.1 on the PSQI, 11.3 ± 6.2 on the ISI, and 6.4 ± 4.2 on the ESS. A higher CUDIT-R was associated with higher PSQI (β = 0.09, 95% CI = 0.01, 0.17) and ESS scores (β = 0.16, 95% CI = 0.07, 0.26). Compared to individuals who only use cannabis, individuals with polysubstance use had higher PSQI, ISI, and ESS scores and reported worse sleep quality and less sleep satisfaction.

**Conclusion:**

Sleep quality and satisfaction were most impaired by polysubstance use, whereas hazardous cannabis use increased sleepiness. The combined use of substances is detrimental to sleep health and highlights an area for public health messaging and awareness.

## Highlights

• Greater cannabis dependence linked with worse sleep quality and greater sleepiness.
• Polysubstance use linked with worse sleep satisfaction compared to cannabis alone.
• Polysubstance use linked with worse sleep scale scores.

## 1 Introduction

Cannabis, tobacco, and alcohol are some of the most widely used substances with distinct effects on physical and mental health (Patrick et al., 2023). In the United States, cannabis use has been on the rise since the early 2000s, largely as a consequence of increased legalization and diminishing perceptions of risk (Patrick et al., 2023). Increased availability and normalization of cannabis have likely contributed to the rise in polysubstance use—the contemporaneous use of two or more substances that can occur either simultaneously, at different times, or through combinations of the substances mixed together (Bunting et al., 2024, Shearer et al., 2022). Simultaneous use of alcohol and cannabis is prevalent among young adults (Lee et al., 2022), while the contemporaneous consumption of cannabis and cigarettes has been on the rise in the US over the past two decades (Rubenstein et al., 2024). Due to potential additive, synergistic, or antagonistic interactions across different timescales, polysubstance use can have complex and unpredictable effects that distinguish it from the isolated use of any one substance (Connor et al., 2023). For example, simultaneous use of alcohol and cannabis appears to reinforce high-risk substance use behavior (Goodhines et al., 2025, Graupensperger et al., 2024), including increasing the risk of alcohol-related blackouts (Schuckit et al., 2017). Polysubstance use is also linked to worse mental health outcomes and higher levels of overall psychological distress, anxiety, and depression (Bhalla et al., 2017). However, little is known about how polysubstance use may influence sleep.

Cannabis is often used (Kuhathasan et al., 2021) and promoted (Corroon et al., 2019) as a sleep aid. Observational evidence suggests that frequency of use influences the effect of cannabis on sleep. For example, one study has shown that cannabis use may decrease sleep onset latency, minimize wake after sleep onset, and increase sleep duration that same night, but perhaps at the expense of increased next-day fatigue (Goodhines et al., 2019). However, daily use of cannabis may interfere with the sleep of healthy adults. For example, one study found that greater daily cannabis use was associated with shorter sleep onset latency, lower sleep efficiency, and more nighttime awakenings among young African Americans (Bell et al., 2022). Another study found that young adults who used cannabis daily had worse sleep quality and greater insomnia symptomatology (Conroy et al., 2016). Moreover, individuals who frequently used cannabis (those using ≥ 20 days out of the past 30) were more likely to have shorter (<6hrs) and longer sleep durations (>9hrs) (Diep et al., 2022, Gonzalez et al., 2023) and greater sleep problems (Walsh et al., 2025).

Cigarette consumption has been linked to insomnia, longer sleep onset latency, shorter sleep duration, and increased daytime sleepiness (Cohrs et al., 2014). Contemporaneous consumption of cannabis products and cigarettes is common (Chu et al., 2023), with evidence suggesting that cigarette dependence is more common among individuals who use cannabis daily versus those who use cannabis less frequently or not at all (Weinberger et al., 2023). Limited studies examining those who use both cannabis and cigarettes show that such people have higher odds of short or long sleep durations (Pan et al., 2023).

Individuals commonly report using alcohol as a sleep aid, with use particularly prevalent among individuals with insomnia (Roehrs and Roth, 2018). Like cannabis, alcohol consumption has been shown to decrease sleep onset latency and consolidate the first half of sleep by reducing wake after sleep onset (Arnedt et al., 2011). However, alcohol negatively affects the second half of sleep via increased nighttime awakenings and disruption of sleep architecture, the cyclical pattern and length of sleep stages (e.g., rapid eye movement) during a sleep period, resulting in an overall reduction of sleep efficiency and increased sleepiness the morning following use (Arnedt et al., 2011). Studies of simultaneous use of alcohol and cannabis indicate possible, proximal benefits when using both substances. Studies of daily alcohol and cannabis use have shown that cannabis attenuated the negative effects of alcohol on sleep (Sznitman et al., 2023, Wycoff et al., 2024), with fewer insomnia symptoms, yet poorer perceived cognitive functioning, compared to days during which neither substance was consumed (Graupensperger et al., 2021). Few studies have considered how different combinations of substance use may affect sleep health across multiple dimensions.

The RU-SATED model has been proposed to capture multiple key dimensions of sleep health, namely RegUlarity, Satisfaction, Alertness, Timing, Efficiency, and Duration (Buysse, 2014). This model of sleep health has demonstrated predictive value for several health outcomes, including morbidity and mortality. One recent study that assessed the separate effects of substance use on multidimensional sleep health found that cannabis and combustible tobacco use was associated with worse sleep health but alcohol consumption was not (Horvat Davey et al., 2024). However, this study only assessed each substance separately and did not consider polysubstance use.

Using a sample of individuals who currently use cannabis, this study aimed to assess the negative effects of cannabis use specifically and how combinations of cannabis use with other substances, namely contemporaneous use with cigarettes and/or alcohol, are associated with multidimensional sleep health over the past month. Based on prior studies, we hypothesized that patterns of regular use of all three substances would be associated with worse sleep satisfaction, lower alertness, lower sleep efficiency, and shorter sleep duration.

## 2 Methods

### 2.1 Participants and recruitment

From May 2017 to December 2018, we recruited 518 adults (18–79 years of age; 35.2 ± 13.4 years old; 65% female) residing in the United States who reported regular cannabis use via online advertisements (e.g., ResearchMatch, Craigslist, X), and flyers posted in public locations and cannabis dispensaries. Posts on Twitter were disseminated via the Oregon Institute of Occupational Health’s official account using targeted hashtags (e.g., #cannabis, #marijuana, #insomnia) to reach individuals searching for related topics. Eligible participants were directed to an informed consent page. After providing consent, respondents completed a 15-minute survey. Upon completion, participants had the option to enter a drawing for a $50 gift certificate (with a 1 in 25 chance of receiving the incentive). The study protocol (#17149) was reviewed and approved by the Oregon Health & Science University Institutional Review Board.

### 2.2 Survey Design

Survey responses were collected and managed using Research Electronic Data Capture (REDCap) tools hosted at Oregon Health & Science University (Harris et al., 2019, Harris et al., 2009). REDCap is a secure clinical and translational research application for building and managing HIPAA- and IRB-compliant online surveys and databases. To ensure response fidelity, REDCap audit trails, survey completion times, and internal consistency across overlapping measures were reviewed to identify potential duplicate, automated, or inattentive responding. Qualifying participants were at least 18 years of age, non-pregnant, living in the United States, used cannabis for a sleep complaint, and used cannabis within the last 24 h.

### 2.3 Cannabis use

Hazardous cannabis use was characterized using the Cannabis Use Disorder Identification Test-Revised (CUDIT-R) (Adamson et al., 2010) This mini-survey consists of eight items querying the frequency and impact of cannabis use. Scores were calculated based on responses, with higher scores indicating a greater likelihood of cannabis use disorder.

### 2.4 Substance use combinations

Cigarette and alcohol use were each assessed by asking participants “Do you smoke cigarettes?” and “Do you drink alcohol?” Possible responses were “every day”, “some days”, or “not at all”. Use status of each substance was defined as 1 if “every day” or “some days” and 0 if “not at all”. Next, substance use groups were defined as “cigarette and cannabis use” if only cigarette use was reported, “alcohol and cannabis use” if only alcohol use was reported, and “cannabis only” if neither cigarette nor alcohol use was reported, because all participants used at least cannabis. If both cigarette and alcohol use were reported, we described the group as “polysubstance use”. The absence of temporal specificity within each survey item precludes the ability to assume simultaneous or sequential use of each substance within the same episode (Bunting et al., 2024, Shearer et al., 2022). Accordingly, polysubstance use in this study reflects the general, contemporaneous substance usage pattern of the individual, rather than the simultaneous use within the same day or episode, or the acute pharmacologic interaction between simultaneous or sequential consumption of substances.

### 2.5 The Pittsburgh sleep quality Index (PSQI)

The PSQI is a 19-item questionnaire that assesses self-rated sleep over the prior month. The scale generates seven component scores (range 0–3, max score of 21 with higher scores indicating worse sleep) that correspond to habitual sleep latency, duration, efficiency, disturbances, quality, use of sleep medications, and daytime dysfunction. A global score ≥ 6 indicates poor sleep quality, discriminating between “good” and “poor” sleepers (Buysse et al., 1989).

Select items from the PSQI (Supplementary Table 1) were used to capture the SATED dimensions of the RU-SATED model. Satisfaction of sleep was assessed by the question: “During the past month, how would you rate your sleep quality overall?” Responses ranged from 0 (“very good”) to 3 (“very bad”). Alertness was assessed by the question: “During the past month, how often have you had trouble staying awake while driving, eating meals, or engaging in social activity?”. Response items were of 0 (“not during the past month”), 1 (“less than once a week”), 2 (“once or twice a week”), and 3 (“three or more times a week”). Timing of bedtime and waketime were respectively assessed by the questions: “During the past month, when have you usually gone to bed?” and “During the past month, what time have you usually gotten up in the morning?”. Efficiency was calculated as the ratio of “During the past month, how many hours of actual sleep did you get per night?” to “During the past month, how many hours were you in bed per night?”, multiplied by 100. Duration was assessed by “During the past month, how many hours of actual sleep did you get per night?”.

### 2.6 Insomnia severity Index (ISI)

The ISI is a seven-item self-report questionnaire, assessing the nature, severity, and impact of insomnia in the past month yielding a score range of 0–28. A score of 0–7 indicates no insomnia symptoms, 8–14 indicates subthreshold/mild insomnia, 15–21 indicates moderate insomnia, and a score > 21 indicates severe insomnia (Morin et al., 2011). The select item of “How satisfied/dissatisfied are you with your current sleep pattern?” was additionally used to assess the dimension of sleep satisfaction, with responses ranging from 0 (“very satisfied”) to 4 (“very dissatisfied”).

### 2.7 The Epworth sleepiness scale (ESS)

The ESS is an 8-item questionnaire assessing the level of daytime sleepiness. Participants rate their likelihood of falling asleep or dozing off on a scale of 0 (“none”) to 3 (“high chance”) for eight different situations that may induce sleepiness (e.g., reading, eating a meal). A score of ≥ 10 indicates daytime sleepiness, and ≥ 18 indicating severe daytime sleepiness (Johns, 1991).

### 2.8 Covariates

Sociodemographic factors and other variables were selected *a priori* due to known associations with sleep health (Grandner et al., 2015, Grandner et al., 2013) and included as covariates. This included age, birth sex (female or male), race (White, Black or African American, or Other), ethnicity (Hispanic or Latino or non-Hispanic/Latino), and highest level of educational attainment (less than high school, high school graduate, some college/associates degree, 4-year college graduate, and graduate school; less than high school responses were merged with high school graduate). Two dummy variables were created for identifying as White or identifying as Black or African American to enable clear interpretation of the association of those races compared to all other racial groups. Other covariates included body type (underweight, average, or overweight; underweight responses was merged with average body type), if they consider themselves an anxious person when not consuming cannabis (strongly disagree, disagree, neither agree nor disagree, agree, or strongly agree), and whether in the past month they took prescribed or “over the counter” medication to help them sleep (“not during the past month”, “less than once a week”, “once or twice a week”, or “three or more times a week” (Buysse et al., 1989); any medication use was merged into a single “yes” response). Age (centered at 18 years old) and self-reported anxiety (centered around “neither agree nor disagree”) were treated as continuous variables. All other covariates were treated as categorical variables.

### 2.9 Statistical analysis

Linear regression models were used to examine the cross-sectional associations of CUDIT-R and substance use classification with the PSQI, ISI, and ESS scales and individual items from those scales. Scores on the CUDIT-R, PSQI, ISI, and ESS scales were all normally distributed (skewness<|2|; kurtosis<|4|). Logistic regression models were used to assess whether CUDIT-R and substance use classification were associated with greater odds of poor sleep quality (PSQI ≥ 6), moderate or severe insomnia (ISI ≥ 15), and daytime sleepiness (ESS ≥ 10). Categories of moderate (ISI ≥ 15) and severe insomnia (ISI > 21), as well as daytime (ESS ≥ 10) and severe daytime sleepiness (ESS ≥ 18), were combined to facilitate interpretation and align with established clinical thresholds indicating minimally yet clinically meaningful impairment. Final models included age, birth sex, race, ethnicity, educational attainment, body type, anxiety when not consuming cannabis, and use of sleep medication. All covariates were entered into the models simultaneously to estimate adjusted associations between the substance use variables of interest and sleep outcomes. Missing data were handled using complete case analysis, with missing observations excluded by default from the analysis. Standardized beta coefficients and 95% confidence intervals (CI) were generated using the “smart” method from the “effectsize” R package (Ben-Shachar et al., 2020) and are reported to aid comparison across linear regression models, whereas odds ratios and 95% CI are reported for logistic regression models. Regression assumptions were evaluated by inspecting residual plots for linearity and homoscedasticity, normality of residuals, and variance inflation factors to assess multicollinearity. No major violations were observed. To account for potential type I errors, we applied false discovery rate (FDR) correction using the Benjamini-Hochberg procedure within families of related outcomes: Continuous sleep scales, clinical thresholds of sleep scales, and dimensions of sleep health. Alpha < 0.05 (two-sided) was used to determine statistical significance.

## 3 Results

Participants reported using cannabis monthly (5%), weekly (32%), daily (63%); smoking cigarettes some days (13%) or every day (17%); and drinking alcohol some days (61%) or every day (3%). Among substance use groups, 25% used only cannabis, 12% used cigarettes and cannabis, 45% used alcohol and cannabis, and 19% used all three substances (i.e., polysubstance use). Average scores were 10.4 ± 5.7 on the CUDIT-R, with 28% having scores indicating hazardous cannabis use (CUDIT-R ≥ 8) and 34% indicating possible cannabis use disorder (CUDIT-R ≥ 12). Sociodemographic information, missingness, and how each covariate relates to substance use in the sample are shown in Table 1. Older age was associated with lower CUDIT-R scores (β = -0.17, 95% CI = -0.27, −0.07) and 3% lower odds of dual alcohol and cannabis use compared to other combinations of substance use (OR = 0.97, 95% CI = 0.96, 0.99). Compared to individuals who completed up to a high school education, individuals with some college education or more were more than twice as likely to use alcohol with cannabis (OR ≥ 2.09, 95% CI = 1.16, 20.90) and individuals who completed college or graduate school were at least 68% less likely to use cigarettes with cannabis (OR ≤ 0.32, 95% CI = 0.05, 0.63).

**Table 1:** **Sample description and associations between covariates and substance use.** Mean ± standard deviation (SD) is shown for age and proportion (%) is shown for other covariates.

**Predictor (N = 518)**	**Mean ± SD or percentage**	**Missingness**	**CUDIT-R**	**Alcohol** **& cannabis**	**Cigarettes& cannabis**	**Alcohol, cigarettes& cannabis**
		N	β [95% CI]	OR [95% CI]	OR [95% CI]	OR [95% CI]
**Age**	35.2 ± 13.4	0	−**0.17 [-0.27, −0.07]**	**0.97 [0.96, 0.99]**	1.01 [1.00, 1.03]	0.99 [0.97, 1.01]
**Birth sex**						
ref: Female	65.1%	3	−	−	−	−
Male	34.9%		**0.10 [0.01, 0.19]**	**1.61 [1.04, 2.51]**	0.76 [0.49, 1.18]	0.88 [0.53, 1.46]
**Race**						
White	77.8%	0	−0.04 [-0.15, 0.07]	0.86 [0.46, 1.57]	1.59 [0.83, 3.20]	1.22 [0.60, 2.72]
Black or African American	6.8%		0.06 [-0.04, 0.17]	1.03 [0.40, 2.73]	2.50 [0.96, 6.66]	2.19 [0.75, 6.39]
**Ethnicity**						
Hispanic or Latino	10.9%	21	−0.08 [-0.16, 0.03]	0.88 [0.47, 1.09]	**0.32 [0.12, 0.70]**	0.53 [0.19, 1.23]
**Education**						
Less than high school	1.9%	1	−	−	−	−
ref: High school graduate	10.8%					
Some college/associates degree	47.4%		−0.04 [-0.33, 0.25]	**2.09 [1.16, 3.81]**	0.59 [0.32, 1.08]	1.06 [0.53, 2.27]
4-year college graduate	24.7%		−0.19 [-0.50, 0.13]	**3.98 [2.06, 7.82]**	**0.32 [0.18, 0.63]**	0.80 [0.37, 1.81]
Graduate school	12.2%		−0.31 [-0.68, 0.07]	**8.17 [3.43, 20.90]**	**0.13 [0.05, 0.32]**	0.56 [0.19, 1.55]
**Body type**						
Underweight	4.1%	1	−	−	−	−
ref: Average	60.5%					
Overweight	35.4%		−0.04 [-0.13, 0.05]	0.71 [0.47, 1.09]	0.92 [0.59, 1.42]	1.08 [0.65, 1.78]
**Anxiety**						
Strongly disagree	14.0%	2	**0.12 [0.03, 0.22]**	1.08 [0.92, 1.27]	1.16 [0.98, 1.38]	**1.23 [1.01, 1.50]**
Disagree	15.9%					
Neither agree nor disagree	21.1%					
Agree	32.0%					
Strongly agree	17.1%					
**Medication to sleep**						
ref: Not during past month	63.0%	5	−	−	−	−
Less than once a week	11.5%		−0.05 [-0.14, 0.04]	0.78 [0.52, 1.18]	1.17 [0.77, 1.78]	0.94 [0.57, 1.51]
Once or twice a week	9.8%					
Three or more times a week	15.8%					

**CUDIT-R**	**Cannabis only**	**Alcohol** **& cannabis**	**Cigarettes& cannabis**	**Alcohol, cigarettes& cannabis**
**Low-risk or non-problematic use (0**–**7)**	52 (27.2%)	88 (46.1%)	22 (11.5%)	28 (14.7%)
**Hazardous cannabis use (8**–**11)**	36 (25.7%)	59 (42.1%)	21 (15.0%)	24 (17.1%)
**Possible cannabis use disorder (12**–**32)**	36 (20.9%)	78 (45.3%)	19 (11.0%)	39 (22.7%)

Note. Standardized beta coefficients [95% CI] for the Cannabis Use Disorders Identification Test–Revised (CUDIT-R) score were estimated with multivariable linear regression models, accounting for all other covariates. Adjusted ORs for drinking alcohol and/or smoking cigarettes some days or every day, in addition to using cannabis, were estimated with multivariable logistic regression models, accounting for all other covariates. Statistically significant results are in **bold** (p < 0.05).

Average scores on the sleep scales were 8.0 ± 4.1 on the PSQI, 11.3 ± 6.2 on the ISI, and 6.4 ± 4.2 on the ESS. Half had good quality sleep, 28% were satisfied with their sleep, 76% had no difficulty staying awake. Average bedtime was 00:54 ± 04:49, waketime was 07:43 ± 02:31, sleep efficiency was 84 ± 14%, and sleep duration was 6.9 ± 1.5 h per night. As shown in Supplementary Table 2, males had lower odds of poor sleep quality (PSQI ≥ 6; OR = 0.58, 95% CI = 0.37, 0.90) or moderate or severe insomnia (ISI ≥ 15; OR = 0.61, 95% CI = 0.39, 0.96) and lower scores on the ISI (β = -0.10, 95% CI = -0.18, −0.01). Identifying as Black or African American was associated with higher ISI scores (β = 0.15, 95% CI = 0.05, 0.25) and greater odds of moderate or severe insomnia (OR = 3.08, 95% CI = 1.18, 8.30). Being overweight was associated with higher scores on the PSQI and ISI (β ≤ 0.11, 95% CI = 0.00, 0.19), moderate or severe insomnia (OR = 1.68, 95% CI = 1.09, 2.61), and daytime sleepiness (ESS ≥ 10; OR = 2.13, 95% CI = 1.23, 3.69). Self-reported anxiety was associated with higher scores on the PSQI and ISI (β ≤ 0.14, 95% CI = 0.04, 0.23) and poor sleep quality (OR = 1.28, 95% CI = 1.07, 1.53). Using medications to promote sleep was associated with higher scores on the PSQI (β = 0.44, 95% CI = 0.36, 0.51) and ISI (β = 0.20, 95% CI = 0.11, 0.28), poor sleep quality (OR = 6.69, 95% CI = 3.95, 11.89), and moderate or severe insomnia (OR = 2.07, 95% CI = 1.37, 3.15).

**Fig. 1:**
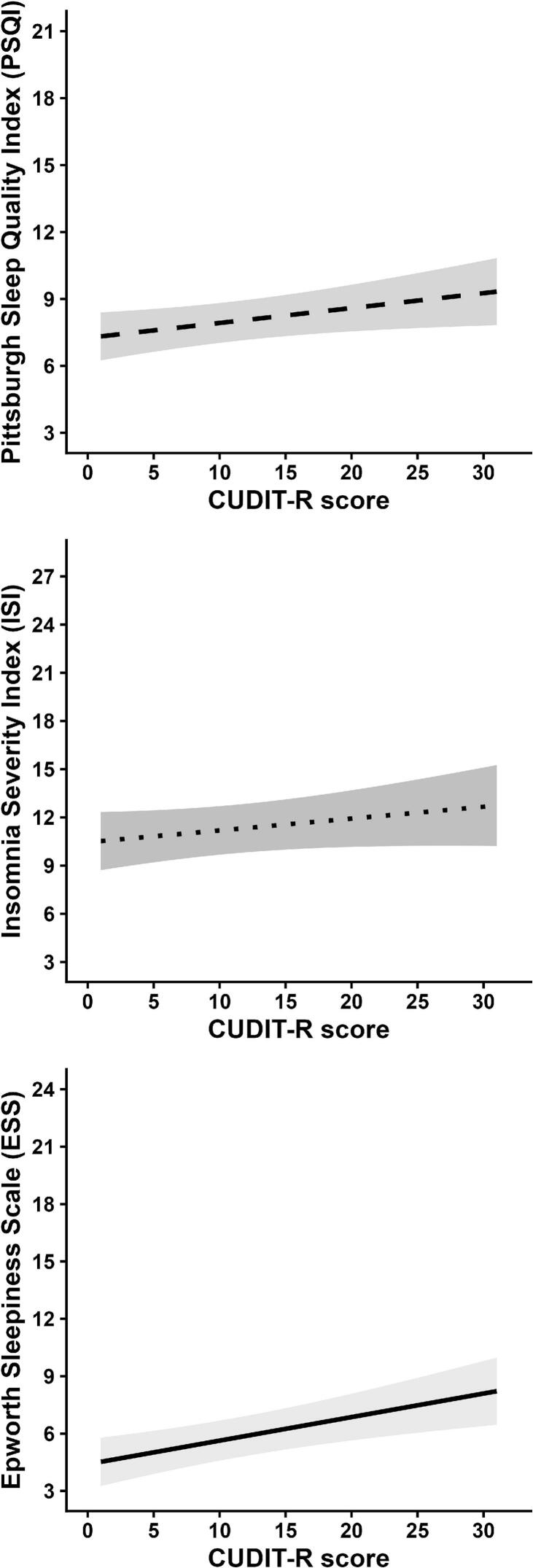
Associations between higher scores on the Cannabis Use Disorders Identification Test–Revised (CUDIT-R) and predicted scores on the Pittsburgh Sleep Quality Index (PSQI; range 0–21; ≥6 indicates poor sleep quality), Insomnia Severity Index (ISI; range 0–28; ≥15 indicates moderate or severe insomnia), and Epworth Sleepiness Scale (ESS; range 0–24; ≥10 indicates daytime sleepiness), accounting for all covariates.

Associations between substance use and sleep are shown in Table 2. As shown in Fig. 1, a higher CUDIT-R was associated with higher scores on the PSQI (β = 0.09, 95% CI = 0.01, 0.17) and ESS (β = 0.16, 95% CI = 0.07, 0.26), even after FDR correction (p_FDR_ ≤ 0.045). Compared to individuals who only use cannabis, individuals with polysubstance use had higher PSQI (β = 0.29, 95% CI = 0.05, 0.53), ISI (β = 0.41, 95% CI = 0.11, 0.71), and ESS scores (β = 0.38, 95% CI = 0.06, 0.70), even after FDR correction (p_FDR_ = 0.020), and were more likely to have moderate or severe insomnia (OR = 2.37, 95% CI = 1.29, 4.43, p_FDR_ = 0.021). There were no associations between CUDIT-R and dimensions of sleep health. Shown in Supplementary Fig. 1, individuals with polysubstance use reported worse sleep satisfaction overall as indicated by worse sleep quality (β = 0.45, 95% CI = 0.18, 0.71, p_FDR_ = 0.007) and less satisfaction with their sleep pattern (β = 0.41, 95% CI = 0.12, 0.70, p_FDR_ = 0.022). Neither polysubstance use nor other substance use groups was associated with other sleep health dimensions.

**Table 2:** Associations of substance use with sleep scales and dimensions of sleep health.

**Substance use**	**PSQI**	**PSQI (≥6)**	**ISI**	**ISI (≥15)**	**ESS**	**ESS (≥10)**	
**(ref = cannabis only)**	β [95% CI]	OR [95% CI]	β [95% CI]	OR [95% CI]	β [95% CI]	OR [95% CI]	
**Cigarette & cannabis use**	0.21 [-0.06, 0.49]	1.85 [0.82, 4.37]	0.22 [−0.13, 0.57]	0.71 [0.32, 1.53]	0.09 [−0.28, 0.46]	1.05 [0.40, 2.61]	
**Alcohol & cannabis use**	0.03 [-0.17, 0.23]	1.23 [0.70, 2.17]	−0.08 [-0.33, 0.17]	0.70 [0.40, 1.22]	0.14 [−0.13, 0.41]	0.86 [0.43, 1.77]	
**Polysubstance use**	**0.29 [0.05, 0.53]**	1.28 [0.65, 2.54]	**0.41 [0.11, 0.71]**	**2.37 [1.29, 4.43]**	**0.38 [0.06, 0.70]**	1.30 [0.60, 2.83]	
**CUDIT-R**	**0.09 [0.01, 0.17]**	1.03 [0.99, 1.08]	0.07 [−0.02, 0.15]	1.02 [0.98, 1.06]	**0.16 [0.07, 0.26]**	1.04 [1.00, 1.10]	

	**Satisfaction**		**Alertness**	**Timing**		**Efficiency**	**Duration**
**Substance use**	**Overall sleep quality**	**Satisfaction with sleep pattern**	**Trouble staying awake**	**Bedtime**	**Waketime**	Duration / time in bed * 100	**Hours of sleep per night**
**(ref = cannabis only)**	β [95% CI]	β [95% CI]	β [95% CI]	β [95% CI]	β [95% CI]	β [95% CI]	β [95% CI]
**Cigarette & cannabis use**	0.23 [-0.08, 0.54]	0.24 [−0.10, 0.58]	−0.16 [−0.52, 0.20]	0.02 [−0.23, 0.26]	0.20 [−0.06, 0.47]	−0.11 [−0.42, 0.20]	−0.32 [−0.67, 0.03]
**Alcohol & cannabis use**	0.16 [-0.06, 0.39]	0.13 [−0.12, 0.38]	−0.01 [−0.28, 0.25]	−0.02 [−0.20, 0.16]	0.11 [−0.08, 0.31]	0.08 [−0.15, 0.30]	0.09 [−0.16, 0.35]
**Polysubstance use**	**0.45 [0.18, 0.71]**	**0.41 [0.12, 0.70]**	0.03 [−0.28, 0.34]	−0.02 [−0.24, 0.19]	0.10 [−0.13, 0.34]	−0.13 [−0.39, 0.14]	−0.26 [−0.56, 0.04]
**CUDIT-R**	0.04 [−0.06, 0.13]	0.04 [−0.05, 0.13]	0.09 [0.00, 0.18]	0.05 [−0.04, 0.14]	0.06 [−0.04, 0.15]	−0.02 [−0.11, 0.07]	−0.02 [−0.11, 0.07]

Note. Standardized beta coefficients [95% CI] for each sleep scale and individual item were estimated with multivariable linear regression models, accounting for all covariates. Adjusted ORs for poor sleep quality (Pittsburgh Sleep Quality Index [PSQI] ≥ 6), moderate or severe insomnia (Insomnia Severity Index [ISI] ≥ 15), and daytime sleepiness (Epworth Sleepiness Scale [ESS] ≥ 10) were estimated with multivariable logistic regression models, accounting for all covariates. The Cannabis Use Disorders Identification Test–Revised (CUDIT-R) was used to assess cannabis use severity. P-values were adjusted for multiple comparisons using false discovery rate correction. Statistically significant results after adjustment are shown in **bold** (p < 0.05).

## 4 Discussion

The goal of this study was to identify how contemporaneous use of cannabis with other substances may be associated with dimensions of sleep health. Using a sample of individuals who regularly use cannabis, the present study highlights both the negative association of cannabis use specifically and different combinations of substances with sleep health. We found that higher scores on the CUDIT-R were associated with higher scores on the PSQI and ESS, yet there was no association with any individual item of multidimensional sleep health. This suggests that individuals who are more greatly affected by their current cannabis use may be more likely to report symptoms of worse global sleep quality and greater daytime sleepiness. However, this perceived detriment to sleep, which individuals may or may not associate with their cannabis use, may contradict the prevailing narrative that cannabis use promotes better sleep (Graupensperger et al., 2023). One possible explanation for this discrepancy is the distinction between acute and chronic effects of cannabis use on sleep. While experimental studies suggest that acute cannabis use may reduce sleep latency and alter sleep architecture (Cousens and DiMascio, 1973, Hoddes et al., 1973, Karacan et al., 1976, Walsh et al., 2021), higher CUDIT-R scores likely reflect more frequent and hazardous patterns of use, capturing cumulative effects that may contribute to worse global sleep quality and daytime sleepiness over time. This distinction may help reconcile short-term perceived sleep benefits with poorer overall sleep health observed in naturalistic and longitudinal studies (Diep et al., 2022, Gonzalez et al., 2023, Pacek et al., 2017).

One-quarter of the sample used only cannabis, while nearly half of the sample used cannabis with alcohol and one-fifth used all three substances. Individuals who used all three substances had elevated scores across all three sleep scales, indicating worse global sleep quality, increased insomnia severity, and greater daytime sleepiness compared to cannabis use alone. Although prior research has found that these substances are separately associated with generally worse self-reported and objectively assessed sleep patterns (Arnedt et al., 2011, Cohrs et al., 2014, Diep et al., 2022), these findings suggest that contemporaneous use of all three substances may additively or synergistically disrupt sleep. This finding is supported by another study that found that polysubstance use was associated with worse insomnia than single-drug use in patients accessing drug detoxification services (Roncero et al., 2012).

One possible explanation for the observed association between polysubstance use and poor sleep involves the addictive potential of commonly used substances, including cannabis. Among individuals who use multiple substances, the reinforcing properties of each substance may interact synergistically, promoting continued use to alleviate fatigue, anxiety, or mood disturbance. Diversification of substance use may reflect attempts to counteract tolerance to a single substance, mitigate the undesirable effects of a given singular substance or to enhance the desirable effects of one or multiple substances. This pattern aligns with a self-medication framework, through which individuals use substances to alleviate symptoms associated with sleep complaints, thereby providing temporary relief but ultimately exacerbating sleep disturbance and dependence over time through a feed-forward process of tolerance and escalation (Goodhines et al., 2025). As tolerance develops, individuals may increase the amount or frequency of use and begin incorporating additional substances in an effort to recapture the initial relief previously experienced, further reinforcing the cycle and worsening sleep health, exposing individuals to levels of use that are increasingly harmful to sleep regulation (Goodhines et al., 2025) and contribute to developing or worsening addiction. We postulate that the feed-forward process may be amplified in the context of polysubstance use, where individuals may both escalate and diversify consumption in pursuit of previously experienced benefits. Neurobiological adaptations underlying addiction may further decouple patterns and motivations of use from the recognition of harm, making it increasingly difficult for individuals to associate their substance use with negative sleep outcomes (Zehra et al., 2018).

The strengths of the present study lie in the diverse sample of individuals who regularly use cannabis for sleep, the ability to compare heterogeneity across multiple contemporaneous substance use combinations within this population, and the assessment of multidimensional sleep health using the SATED model. This approach to assessing sleep richly characterizes sleep better than querying sleep duration alone. However, given the cross-sectional nature of the study, we cannot assess temporal precedence, especially because eligibility required use of cannabis for a sleep complaint and use within the last 24 h. Worse sleep health may have preceded the decision to use other substances contemporaneously alongside cannabis, or other substances may have been used to promote sleep. This recruitment strategy likely overrepresents individuals who perceive cannabis as beneficial for sleep or who are motivated to self-medicate sleep complaints. Accordingly, observed associations should be interpreted as reflecting variation in multidimensional sleep health across patterns of contemporaneous substance use within a cannabis use population, rather than effects generalizable to individuals who use cannabis for other reasons or do not use cannabis at all. Moreover, because the study did not include a non-cannabis use comparison group, we cannot determine whether associations with cigarette or alcohol use reflect synergistic effects with cannabis or effects that would also be present in the absence of cannabis. Next, the study did not objectively assess sleep, including the examination of macro- and micro-sleep architecture (Velzeboer et al., 2025). Other unassessed factors may additionally contribute to or mediate the association between substance use and sleep, such as employment, household income, or psychiatric diagnoses. For example, daytime sleepiness has been shown to be influenced by psychiatric disorders, such as anxiety and depression, in addition to substance use (Garey et al., 2020); although we accounted for self-reported anxiety, we did not assess such diagnosed health conditions in the present study and thus were unable to control for them in our analysis. Finally, the analytic sample may overrepresent the proportion of people living in the U.S. who consume alcohol (Substance Abuse and Mental Health Services Administration, 2025), and the study may have been underpowered to assess the effects of small substance use combination groups, namely cigarettes and cannabis use, on multidimensional sleep health. Future studies should address these limitations by leveraging polysomnography and nationally representative multi-site trials to holistically assess sleep architecture (Velzeboer et al., 2025) and the possibility of feed-forward processes (Goodhines et al., 2025) between polysubstance use and sleep health.

Our results underscore the need for targeted public health messaging to address risks associated with polysubstance use, particularly the influence it may have on sleep health. In clinical settings, these findings support the importance of assessing multidimensional sleep health *alongside* substance use behaviors, especially when patients present with concerns in either domain. The effects of substance use on sleep may be overlooked amid other consequences of substance use, such as disruptions to daily functioning or comorbid psychological conditions. However, given the foundational role of sleep in physical and mental health, clinicians should routinely incorporate sleep assessments into substance use evaluations, and vice versa, to better identify and address potential bidirectional influences (Chakravorty et al., 2018). Lastly, our findings endorse further need for research into the interactions between substance use, especially polysubstance use, and important health measures like sleep.

## CRediT authorship contribution statement

**David A. Reichenberger:** Writing – review & editing, Writing – original draft, Visualization, Methodology, Formal analysis, Conceptualization. **Joey Hebl:** Writing – review & editing, Writing – original draft. **Steven A. Shea:** Writing – review & editing, Funding acquisition. **Nicole P. Bowles:** Writing – review & editing, Project administration, Methodology, Investigation, Funding acquisition, Conceptualization.

## Funding

This work was supported by National Heart, Lung, and Blood Institute (NHLBI) NIH grants *K*01 HL151745, R35 HL155681, the National Center for Advancing Translational Sciences (NCATS), National Institutes of Health, through Grant Award Number UL1TR002369, and the Oregon Institute of Occupational Health Sciences at Oregon Health & Science University via funds from the Division of Consumer and Business Services of the State of Oregon (ORS 656.630). DAR was supported by T32 HL166128.

## Declaration of competing interest

The authors declare that they have no known competing financial interests or personal relationships that could have appeared to influence the work reported in this paper.

## Data Availability

Data will be made available on request.
