# Supplementary material for: Associations of cannabis, alcohol, and tobacco use combinations with sleep health
Source: Addict Behav Rep. 2026 Feb 21;23:100680. doi: 10.1016/j.abrep.2026.100680 (PMC12963894; doi:10.1016/j.abrep.2026.100680)
Supplement: Supplementary Data 1 [file mmc1.docx]

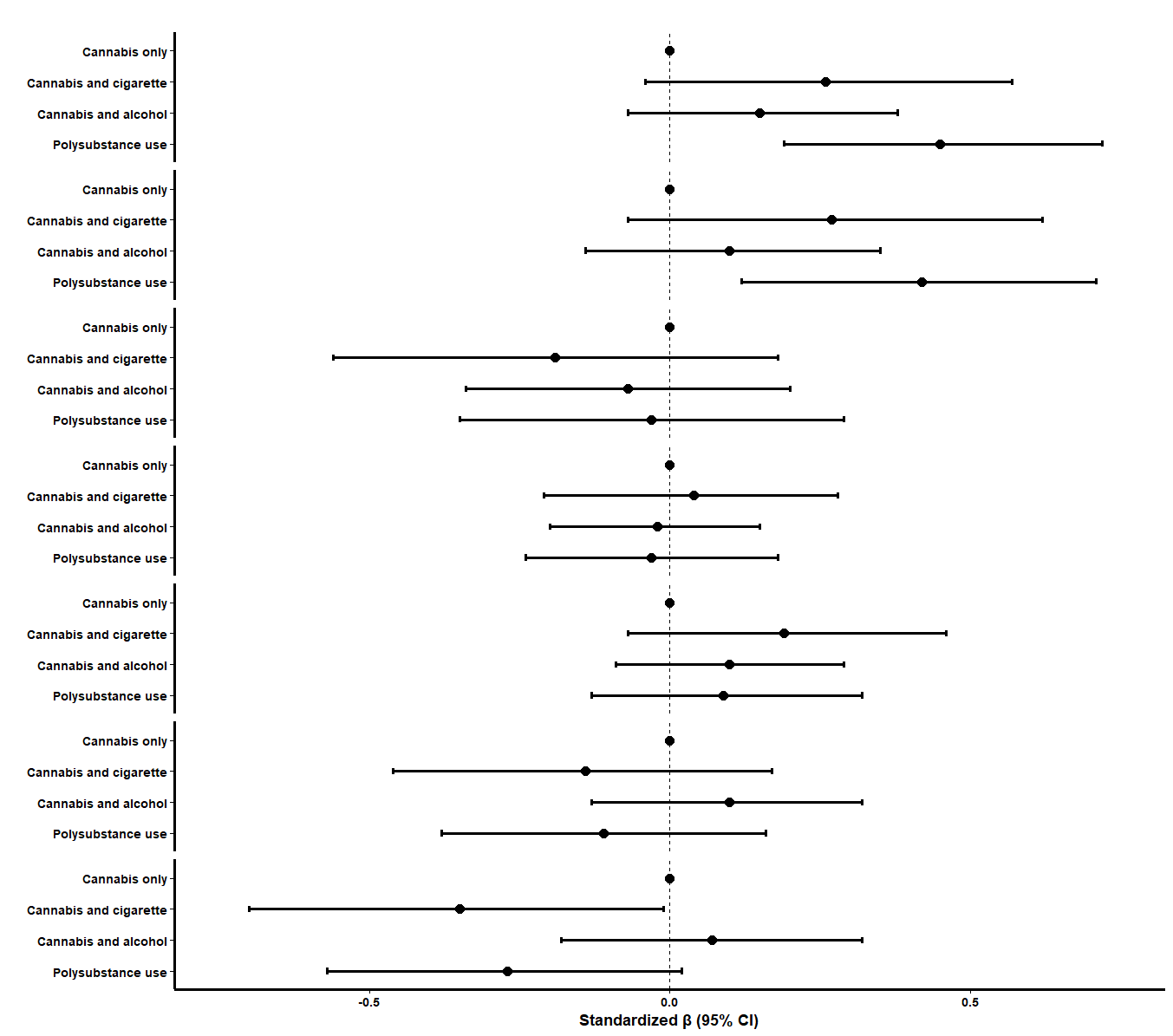


Duration / time in bed * 100

Satisfaction with sleep pattern‡

Hours of sleep per night

Waketime

Bedtime

Trouble staying awake‡

Overall sleep quality‡

Overall sleep quality‡

Overall sleep quality‡

Overall sleep quality‡

Overall sleep quality‡

**Supplementary Figure 1.** Forest plot depicting the standardized βs and 95% CI for each substance use combination compared to the reference category of cannabis only, accounting for all covariates.
‡higher is worse
