# Supplementary material for: Associations of cannabis, alcohol, and tobacco use combinations with sleep health
Source: Addict Behav Rep. 2026 Feb 21;23:100680. doi: 10.1016/j.abrep.2026.100680 (PMC12963894; doi:10.1016/j.abrep.2026.100680)
Supplement: Supplementary Data 2 [file mmc2.docx]

| **Supplementary Table 1:** Sleep health dimensions | | |
| --- | --- | --- |
| Dimension | Items | Scale |
| Satisfaction | During the past month, how would you rate your sleep quality overall? | PSQI |
|  | How satisfied/dissatisfied are you with your current sleep pattern? | ISI |
| Alertness | During the past month, how often have you had trouble staying awake while driving, eating meals, or engaging in social activity? | PSQI |
| Timing | During the past month, when have you usually gone to bed? | PSQI |
|  | During the past month, what time have you usually gotten up in the morning? | PSQI |
| Efficiency | Duration / time in bed * 100 | PSQI |
| Duration | During the past month, how many hours of actual sleep did you get per night? | PSQI |
| Note. PSQI = Pittsburgh Sleep Quality Index; ISI = Insomnia Severity Index | | |

| **Supplementary Table 2: Associations between covariates and sleep scales.** | | | | | | | |
| --- | --- | --- | --- | --- | --- | --- | --- |
| **Predictor (N = 518)** | **PSQI** | **PSQI (≥6)** | **ISI** | **ISI (≥15)** | **ESS** | **ESS (≥10)** |  |
|  | β [95% CI] | OR [95% CI] | β [95% CI] | OR [95% CI] | β [95% CI] | OR [95% CI] |  |
| **Age** | -0.03 [-0.12, 0.05] | 0.98 [0.97, 1.00] | -0.08 [-0.17, -0.02] | 0.99 [0.98, 1.01] | 0.03 [-0.06, 0.13] | 1.00 [0.98, 1.02] |  |
| **Birth sex (ref: Female)** | -0.05 [-0.13, 0.03] | **0.58 [0.37, 0.90]** | **-0.10 [-0.18, -0.01]** | **0.61 [0.39, 0.96]** | -0.07 [-0.16, 0.03] | 0.74 [0.40, 1.31] |  |
| **Race** |  |  |  |  |  |  |  |
| White | -0.00 [-0.10, 0.09] | 1.09 [0.57, 2.07] | 0.03 [-0.08, 0.13] | 0.89 [0.48, 1.68] | -0.02 [-0.12, 0.09] | 0.67 [0.31, 1.52] |  |
| Black or  African American | 0.05 [-0.04, 0.14] | 1.54 [0.52, 4.89] | **0.15 [0.05, 0.25]** | **3.08 [1.18, 8.30]** | 0.04 [-0.07, 0.14] | 1.60 [0.52, 4.87] |  |
| **Ethnicity** |  |  |  |  |  |  |  |
| Hispanic or Latino | -0.03 [-0.11, 0.05] | 0.64 [0.32, 1.29] | -0.02 [-0.11, 0.06] | 1.00 [0.49, 1.96] | -0.09 [-0.18, 0.00] | 0.39 [0.11, 1.05] |  |
| **Education** |  |  |  |  |  |  |  |
| Less than high school | - | - | - | - | - | - |  |
| ref: High school graduate |  |  |  |  |  |  |  |
| Some college/associates degree | 0.18 [-0.07, 0.43] | 1.07 [0.54, 2.07] | 0.14 [-0.17, 0.45] | 1.78 [0.91, 3.65] | 0.21 [-0.08, 0.50] | 1.44 [0.59, 4.06] |  |
| 4-year college graduate | -0.13 [-0.40, 0.14] | 0.76 [0.37, 1.55] | -0.15 [-0.48, 0.19] | 1.16 [0.56, 2.50] | 0.10 [-0.21, 0.41] | 1.57 [0.60, 4.63] |  |
| Graduate school | -0.18 [-0.50, 0.14] | 0.64 [0.28, 1.46] | -0.06 [-0.46, 0.33] | 0.97 [0.38, 2.44] | 0.18 [-0.18, 0.55] | 2.08 [0.68, 6.84] |  |
| **Body type** |  |  |  |  |  |  |  |
| Underweight | - | - | - | - | - | - |  |
| ref: Average |  |  |  |  |  |  |  |
| Overweight | **0.11 [0.03, 0.19]** | 1.48 [0.92, 2.40] | **0.09 [0.00, 0.18]** | **1.68 [1.09, 2.61]** | 0.08 [-0.02, 0.17] | **2.13 [1.23, 3.69]** |  |
| **Anxiety** | **0.12 [0.04, 0.20]** | **1.28 [1.07, 1.53]** | **0.14 [0.05, 0.23]** | 1.18 [1.00, 1.41] | 0.08 [-0.02, 0.17] | 1.07 [0.86, 1.33] |  |
| **Medication to sleep  (ref: Not during past month)** | **0.44 [0.36, 0.51]** | **6.69 [3.95, 11.89]** | **0.20 [0.11, 0.28]** | **2.07 [1.37, 3.15]** | 0.00 [-0.09, 0.09] | 1.62 [0.96, 2.73] |  |
| Note. Standardized beta coefficients [95% CI] for each sleep scale were estimated with multivariable linear regression models, accounting for all covariates. Adjusted ORs for poor sleep quality (Pittsburgh Sleep Quality Index [PSQI] ≥6), moderate or severe insomnia (Insomnia Severity Index [ISI] ≥15), and daytime sleepiness (Epworth Sleepiness Scale [ESS] ≥10) were estimated with multivariable logistic regression models, accounting for all covariates. Statistically significant results are shown in **bold** (p<0.05). | | | | | | | |
